# Supplementary material for: Broadly neutralizing and protective nanobodies against SARS-CoV-2 Omicron subvariants BA.1, BA.2, and BA.4/5 and diverse sarbecoviruses
Source: Nat Commun. 2022 Dec 27;13:7957. doi: 10.1038/s41467-022-35642-2 (PMC9792944; doi:10.1038/s41467-022-35642-2)
Supplement: Supplementary file 1 — Supplementary Information File [file 41467_2022_35642_MOESM1_ESM.pdf]

Supplementary Materials for

Broadly neutralizing and protective nanobodies against SARS-CoV-2 Omicron subvariants BA.1, BA.2, and BA.4/5 and diverse sarbecoviruses

Mingxi Li, Yifei Ren, Zhen Qin Aw, Bo Chen, Ziqing Yang, Yuqing Lei, Lin Cheng, Qingtai Liang, Junxian Hong, Yiling Yang, Jing Chen, Yi Hao Wong, Jing Wei, Sisi Shan, Senyan Zhang, Jiwan Ge, Ruoke Wang, Jay Zengjun Dong, Yuxing Chen, Xuanling Shi, Qi Zhang, Zheng Zhang, Justin Jang Hann Chu*, Xinquan Wang*, Linqi Zhang*

*Corresponding author. Email: miccjh@nus.edu.sg, xinquanwang@tsinghua.edu.cn, zhanglinqi@tsinghua.edu.cn

This PDF file includes:

Figs. S1 to S6
Tables S1 to S4

Figure S1

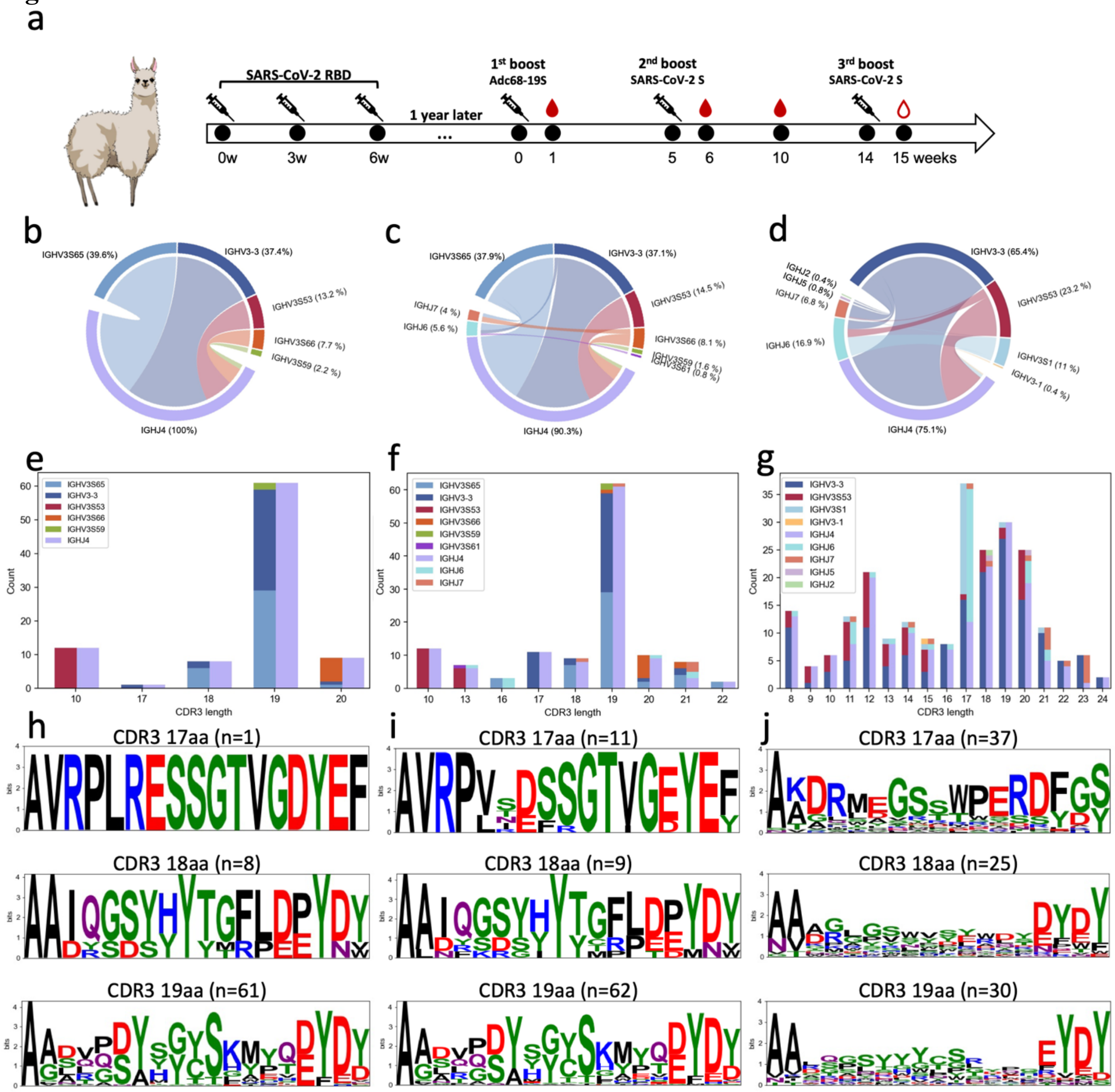

Fig. S1. Genetic characterization and comparison between isolated and published nanobodies against SARS-CoV-2. (a) Immunization schedule for alpaca. An alpaca was immunized with three-time subcutaneous injections of 200 μg recombinant RBD of prototype SARS-CoV-2 in Freund adjuvant. About one year later, one-time subcutaneous injection of 10^{11} viral particle AdC68-19S vaccine and two-time subcutaneous injections of 200 μg recombinant S-2P protein of prototype SARS-CoV-2 in Freund adjuvant were carried out. Serum samples were collected at 1, 6 and 10 weeks after AdC68-19S immunization to monitor the titer of specific antibody. One week after the last immunization, blood samples were collected for library construction. **(b, c, d)** Chord diagrams comparing the V and J gene segments usage and pairing among the 91 cross-neutralizing, total 124 isolated, and published 237 nanobodies in the CoV-AbDab database. Each V and J segments are colored and indicated around the peripheral circle together with their percentage among the total number of nanobodies analyzed. V/J pairs are linked

by colored arcs, and the size of which is proportional to the total number of nanobodies analyzed. **(e, f, g)** The bar plot showing the distribution and proportion of various CDR3 length among the 91 cross-neutralizing, total 124 isolated and 237 published nanobodies. The specific V and J gene usage associated with each CDR3 length are colored and shown. **(h, i, j)** Comparison of CDR3 logo sequence among the 91 cross-neutralizing, total 124 isolated, and 237 published nanobodies, analyzed separately for 17-residue (top), 18-residue (middle), and 19-reside (bottom) CDR3. The number of sequences analyzed for each CDR3 group are indicated.

Figure S2

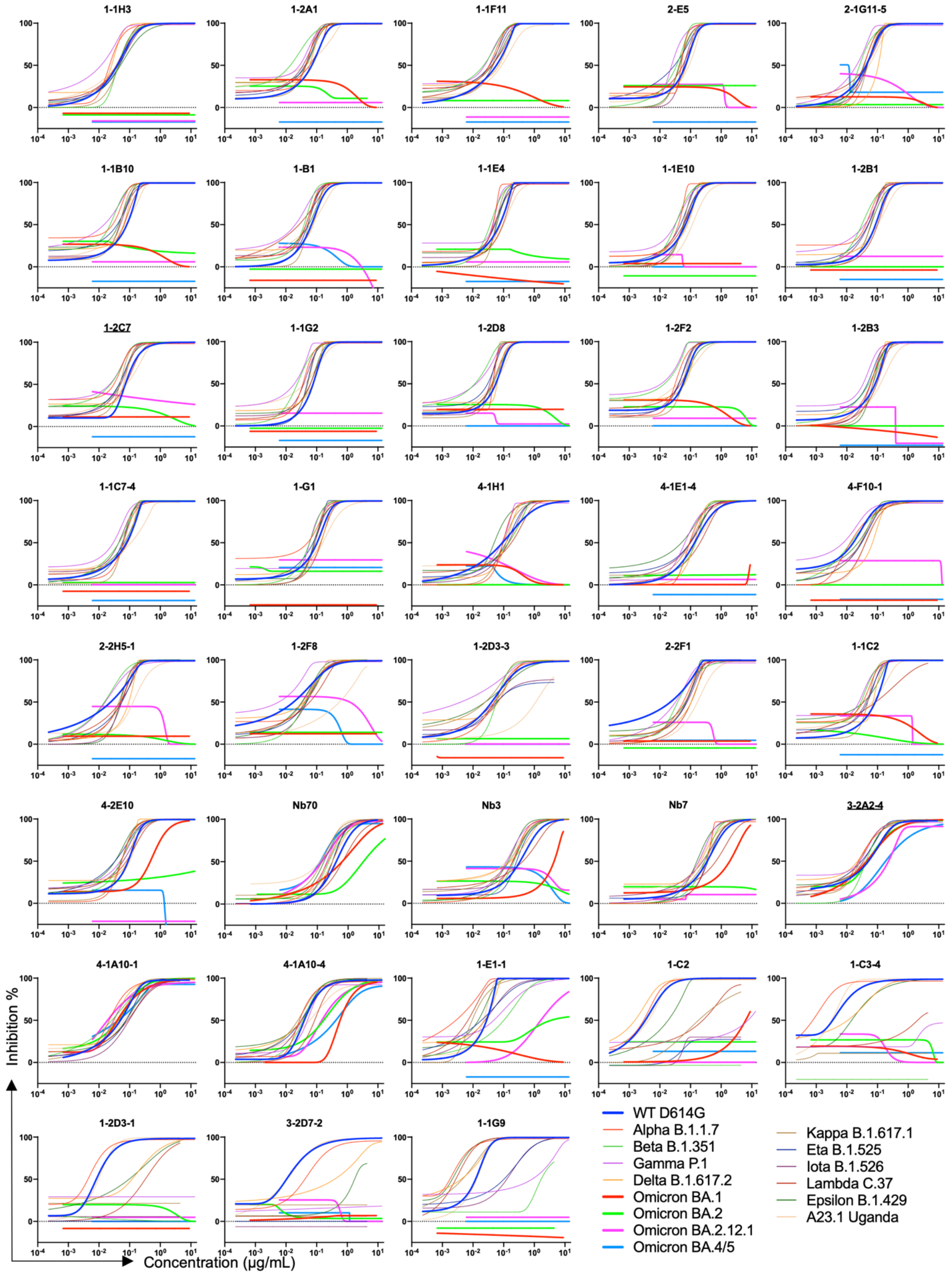
1-1H3
1-2A1
1-1F11
2-E5
2-1G11-5
1-1B10
1-B1
1-1E4
1-1E10
1-2B1
1-2C7
1-1G2
1-2D8
1-2F2
1-2B3
1-1C7-4
1-G1
4-1H1
4-1E1-4
4-F10-1
2-2H5-1
1-2F8
1-2D3-3
2-2F1
1-1C2
4-2E10
Nb70
Nb3
Nb7
3-2A2-4
4-1A10-1
4-1A10-4
1-E1-1
1-C2
1-C3-4
1-2D3-1
3-2D7-2
1-1G9
Inhibition %
Concentration (μg/mL)
WT D614G
Alpha B.1.1.7
Beta B.1.351
Gamma P.1
Delta B.1.617.2
Omicron BA.1
Omicron BA.2
Omicron BA.2.12.1
Omicron BA.4/5
Kappa B.1.617.1
Eta B.1.525
Iota B.1.526
Lambda C.37
Epsilon B.1.429
A23.1 Uganda

Fig. S2. Neutralizing activity of isolated nanobodies against SARS-CoV-2 variants. Serial dilutions of each nanobody were evaluated against pseudoviruses carrying spike protein of prototype and variants of SARS-CoV-2. Neutralizing activity was defined as the percent reduction in luciferase activities compared to no antibody controls. Results presented are representatives of three independent experiments.

Figure S3

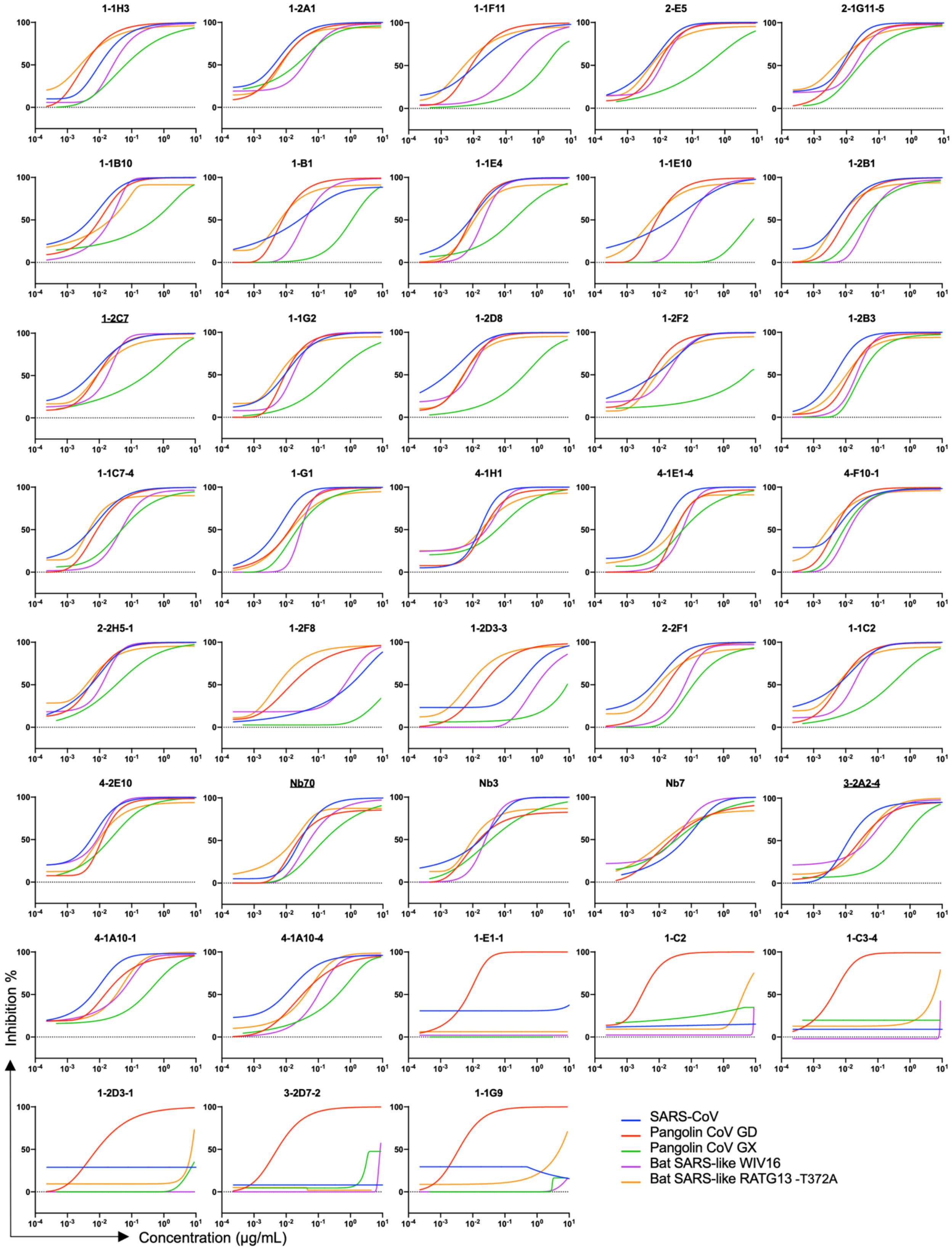
1-1H3
1-2A1
1-1F11
2-E5
2-1G11-5
1-1B10
1-B1
1-1E4
1-1E10
1-2B1
1-2C7
1-1G2
1-2D8
1-2F2
1-2B3
1-1C7-4
1-G1
4-1H1
4-1E1-4
4-F10-1
2-2H5-1
1-2F8
1-2D3-3
2-2F1
1-1C2
4-2E10
Nb70
Nb3
Nb7
3-2A2-4
4-1A10-1
4-1A10-4
1-E1-1
1-C2
1-C3-4
1-2D3-1
3-2D7-2
1-1G9
Inhibition %
Concentration (μg/mL)
SARS-CoV
Pangolin CoV GD
Pangolin CoV GX
Bat SARS-like WIV16
Bat SARS-like RATG13 -T372A

Fig. S3. Neutralizing activity of isolated nanobodies against hACE2-dependent sarbecoviruses. Serial dilutions of each nanobody were tested against pseudoviruses carrying spike protein of various sarbecoviruses. Neutralizing activity was defined as the percent reduction in luciferase activities compared to no antibody controls. Results presented are representatives of three independent experiments.

Figure S4

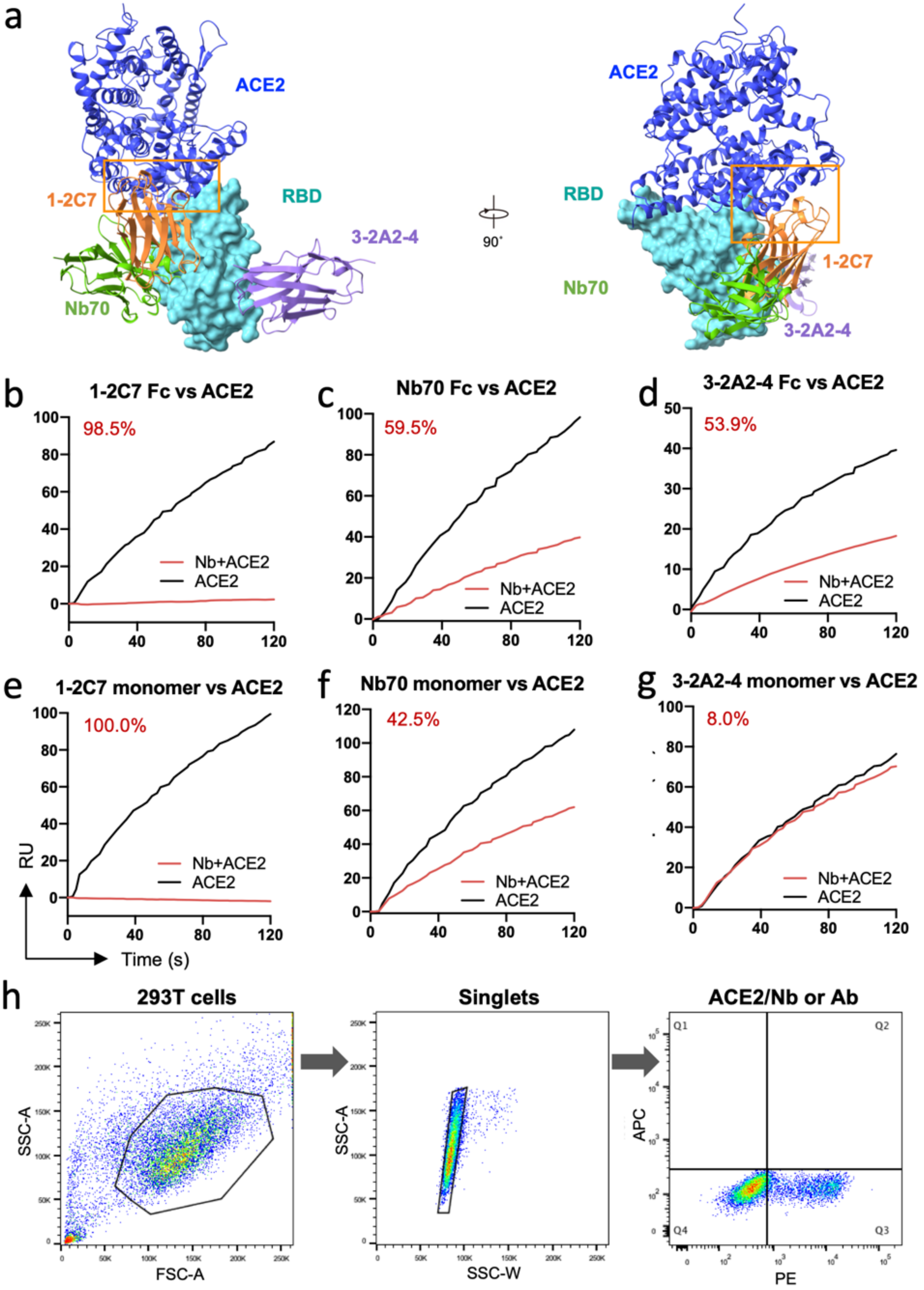
a
ACE2
1-2C7
RBD
3-2A2-4
Nb70
90°
ACE2
RBD
1-2C7
Nb70
3-2A2-4
b
1-2C7 Fc vs ACE2
98.5%
Nb+ACE2
ACE2
c
Nb70 Fc vs ACE2
59.5%
Nb+ACE2
ACE2
d
3-2A2-4 Fc vs ACE2
53.9%
Nb+ACE2
ACE2
e
1-2C7 monomer vs ACE2
100.0%
Nb+ACE2
ACE2
RU
Time (s)
f
Nb70 monomer vs ACE2
42.5%
Nb+ACE2
ACE2
g
3-2A2-4 monomer vs ACE2
8.0%
Nb+ACE2
ACE2
h
293T cells
SSC-A
FSC-A
Singlets
SSC-A
SSC-W
ACE2/Nb or Ab
Q1
Q2
Q4
Q3
APC
PE

Fig. S4. Binding mode of three nanobodies to RBD relative to receptor ACE2 and competition of nanobodies with ACE2 measured by SPR. (a) 1-2C7, Nb70, and 3-2A2-4 were aligned to SARS-CoV-2 RBD-ACE2 complex (PDB: 6M0J). 1-2C7 (orange) clashed with ACE2 through steric hindrance, indicated by the orange box. Nb70 and 3-2A2-4 were distinctive from ACE2 binding and no potential steric clash was noticed. The SARS-CoV-2 RBD is shown in light cyan, Nb70 in green, and 3-2A2-4 in purple. The sensorgrams show distinct binding patterns of ACE2 to prototype SARS-CoV-2 RBD with (red curve) or without (black curve) prior incubation with each testing nanobody in Fc form **(b, d and d)** or in monomer form **(e, f and g)**. The competition capacity of each nanobody is measured by the level of reduction in the response unit comparing with or without prior incubation. Results presented are representatives of two independent experiments. **(h)** The gating strategy for competitive binding of nanobodies with ACE2 to SARS-CoV-2 spike by cell surface staining.

Figure S5

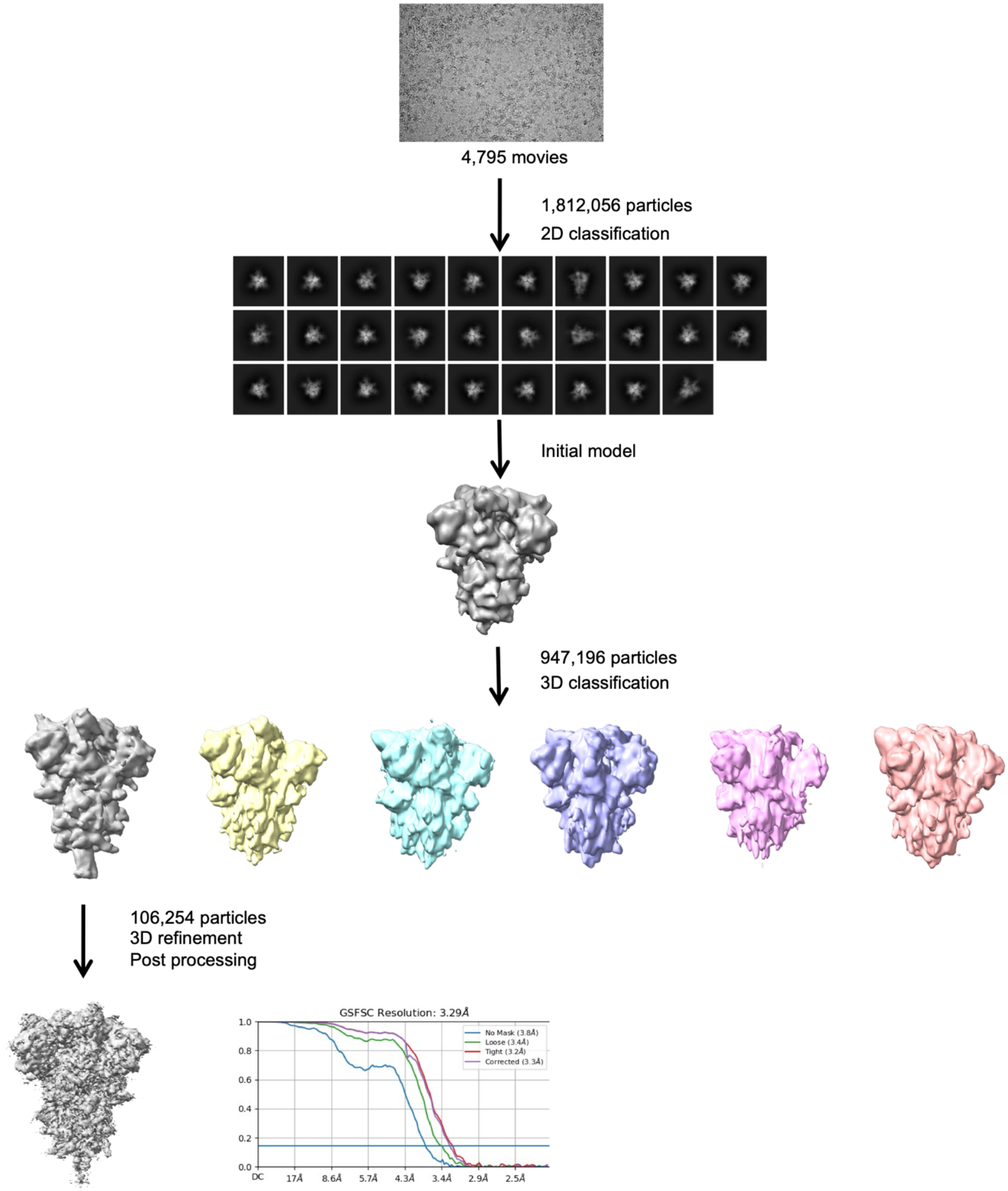

Fig. S5. Processing workflow of the 3-2A2-4 and SARS-CoV-2 Omicron BA.1 Spike complex cryo-EM data. Initially, 4,795 movies were collected and 1,812,056 particles were subjected to 2D classification. After three additional 2D classification, the best selected 947,196 particles were applied for initial model and 3D classification. A subset of 106,254 particle images from 3

nanobodies bound to 3 down RBD were further subject to 3D refinement and post-processing. The final resolution is 3.29 Å.

Figure S6

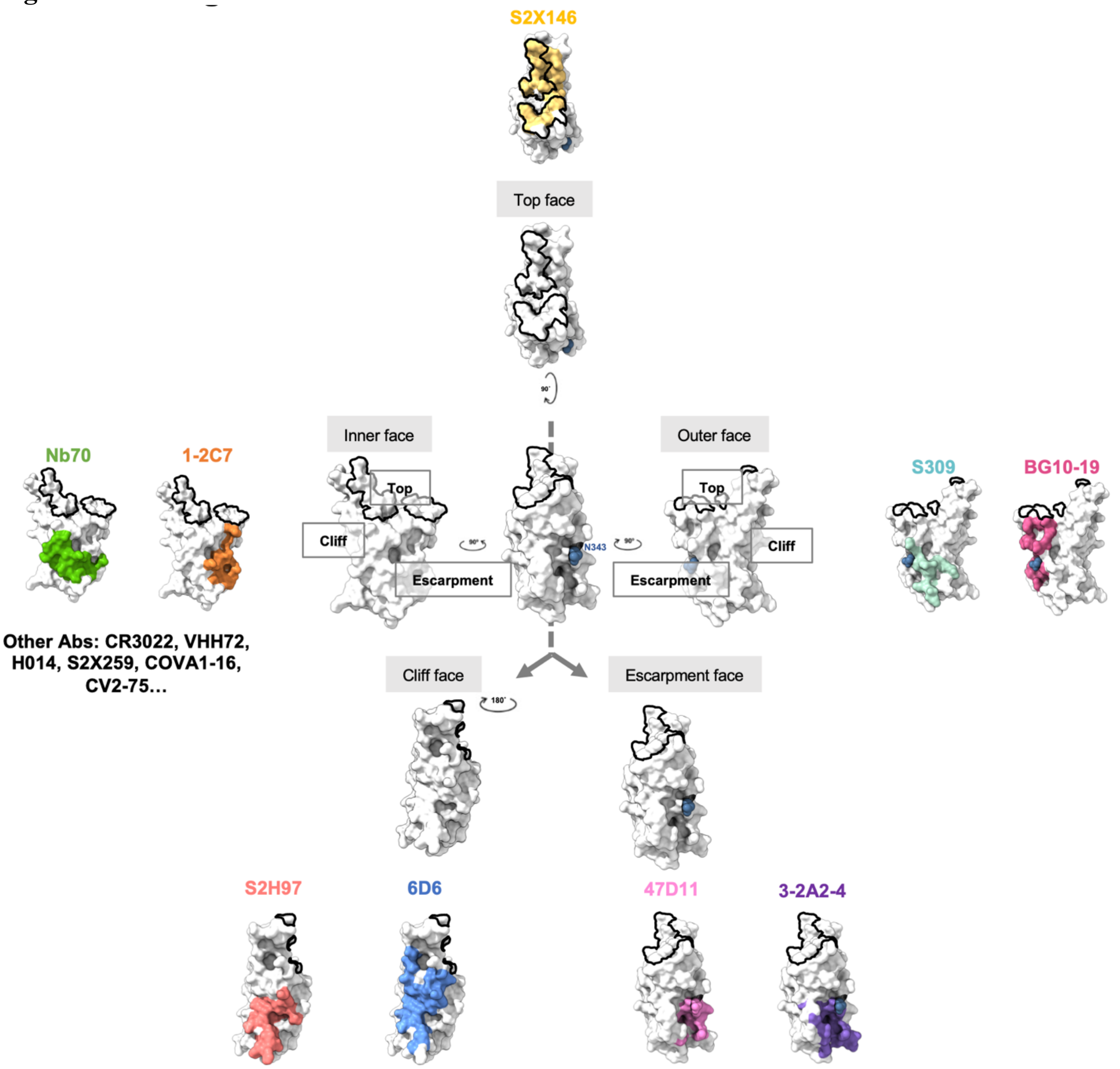

Fig. S6. Structural illustration of SARS-CoV-1 and SARS-CoV-2 cross-neutralizing epitopes recognized by the three representative nanobodies and various published antibodies. Cross-neutralizing epitopes on the top face of RBD is recognized by S2X146, inner face by Nb70 and 1-2C7, outer face by S309 and BG10-19, cliff face by S2H97 and 6D6, and escarpment face by 47D11 and 3-2A2-4. The glycosylation site at position 343 (N343), conserved across the sarbecovirus subgenus, is colored in dark blue. The ACE2-binding site is outlined in black. The view in the middle is the starting view for all others. The middle view in the 3rd row is the starting view for all others.

Table S1. IC50 values of nanobodies with standard deviation against diverse panel of pseudoviruses

IC50±SD (μg/mL)	WT D614G	Alpha B.1.1.7	Beta B.1.351	Gamma P.1	Delta B.1.617.2	Omicron BA.1	Omicron BA.2	Omicron BA.2.12.1	Omicron BA.4/5	Kappa B.1.617.1	Eta B.1.525	Lota B.1.526	Lambda C.37	Epsilon B.1.429	A23.1 Uganda	SARS-CoV-1	Pangolin CoV GD	Pangolin CoV GX	Bat CoV WIV16	Bat CoV RATG13-T372A
1-1H3	0.0659± 0.0123	0.0222± 0.0026	0.0377± 0.0267	0.0129± 0.0079	0.0245± 0.0042	BDL	BDL	BDL	BDL	0.0501± 0.0241	0.0446± 0.0310	0.0498± 0.0291	0.0355± 0.0167	0.0438± 0.0796	0.0378± 0.0088	0.0103± 0.0062	0.0041± 0.0002	0.0508± 0.0292	0.0315± 0.0183	0.0027± 0.0003
1-2A1	0.0543± 0.0118	0.0428± 0.0144	0.0164± 0.0135	0.0229± 0.0215	0.0491± 0.0407	BDL	BDL	BDL	BDL	0.0453± 0.0127	0.0410± 0.0229	0.0358± 0.0079	0.0568± 0.0301	0.0243± 0.0522	0.0601± 0.0233	0.0042± 0.0019	0.0064± 0.0003	0.0286± 0.0104	0.0350± 0.0042	0.0060± 0.0031
1-1F11	0.0405± 0.0157	0.0325± 0.0088	0.0179± 0.0093	0.0211± 0.0147	0.0676± 0.0458	BDL	BDL	BDL	BDL	0.0428± 0.0036	0.0344± 0.0188	0.0452± 0.0112	0.0587± 0.0479	0.0289± 0.0304	0.0868± 0.0011	0.0138± 0.0067	0.0086± 0.0016	1.9179± 0.3809	0.2082± 0.1034	0.0056± 0.0019
2-E5	0.0583± 0.0168	0.0472± 0.0067	0.0349± 0.0203	0.0242± 0.0056	0.0736± 0.0411	BDL	BDL	BDL	BDL	0.0410± 0.0060	0.0392± 0.0067	0.0555± 0.0110	0.0568± 0.0099	0.0449± 0.0027	0.0882± 0.0104	0.0385± 0.0010	0.0080± 0.0012	0.2050± 0.0620	0.0110± 0.0049	0.0054± 0.0001
2-1G11-5	0.0489± 0.0071	0.0378± 0.0051	0.0185± 0.0093	0.0237± 0.0171	0.0876± 0.0041	BDL	BDL	BDL	BDL	0.0466± 0.0118	0.0416± 0.0184	0.0449± 0.0049	0.0434± 0.0138	0.0332± 0.0131	0.0512± 0.0098	0.0701± 0.0026	0.0081± 0.0024	0.0128± 0.0225	0.0163± 0.0051	0.0044± 0.0017
1-1B10	0.0893± 0.0413	0.0389± 0.0207	0.0255± 0.0146	0.0279± 0.0166	0.0500± 0.0299	BDL	BDL	BDL	BDL	0.0446± 0.0018	0.0456± 0.0174	0.0472± 0.0135	0.0577± 0.0222	0.0307± 0.0416	0.0764± 0.0244	0.0052± 0.0012	0.0100± 0.0032	0.4442± 0.1388	0.0245± 0.0133	0.0185± 0.0004
1-B1	0.0450± 0.0242	0.0429± 0.0096	0.0286± 0.0167	0.0215± 0.0127	0.0762± 0.0350	BDL	BDL	BDL	BDL	0.0547± 0.0029	0.0461± 0.0183	0.0408± 0.0172	0.0489± 0.0563	0.0332± 0.0452	0.0680± 0.0021	0.0256± 0.0332	0.0075± 0.0029	0.8716± 0.3706	0.0429± 0.0084	0.0068± 0.0028
1-1E4	0.0516± 0.0159	0.0427± 0.0102	0.0256± 0.0159	0.0319± 0.0296	0.0746± 0.0232	BDL	BDL	BDL	BDL	0.0552± 0.0029	0.0526± 0.0227	0.0474± 0.0058	0.0776± 0.0394	0.0419± 0.0547	0.0896± 0.0335	0.0083± 0.0063	0.0084± 0.0002	0.1122± 0.1188	0.0262± 0.0123	0.0115± 0.0057
1-1E10	0.0383± 0.0239	0.0346± 0.0132	0.0251± 0.0136	0.0313± 0.0223	0.0743± 0.0229	BDL	BDL	BDL	BDL	0.0562± 0.0013	0.0457± 0.0124	0.0675± 0.0298	0.0672± 0.0568	0.0433± 0.0270	0.1170± 0.0215	0.0359± 0.0479	0.0078± 0.0016	5.6497± 273.796 6	0.0913± 0.0309	0.0067± 0.0043
1-2B1	0.0621± 0.0083	0.0410± 0.0164	0.0290± 0.0194	0.0376± 0.0233	0.0781± 0.0166	BDL	BDL	BDL	BDL	0.0587± 0.0053	0.0486± 0.0166	0.0527± 0.0155	0.0922± 0.0242	0.0496± 0.0376	0.1030± 0.0411	0.0055± 0.0030	0.0097± 0.0014	0.0414± 0.0035	0.0423± 0.0001	0.0061± 0.0023
1-2C7	0.0559± 0.0119	0.0280± 0.0008	0.0229± 0.0115	0.0231± 0.0178	0.0532± 0.0362	BDL	BDL	BDL	BDL	0.0489± 0.0104	0.0444± 0.0141	0.0495± 0.0109	0.0746± 0.0209	0.0297± 0.0374	0.0793± 0.0030	0.0064± 0.0060	0.0087± 0.0002	0.3657± 0.1384	0.0175± 0.0006	0.0091± 0.0019
1-1G2	0.0725± 0.0129	0.0362± 0.0162	0.0212± 0.0178	0.0198± 0.0143	0.0719± 0.0442	BDL	BDL	BDL	BDL	0.0510± 0.0065	0.0475± 0.0136	0.0357± 0.0040	0.0613± 0.0116	0.0444± 0.0540	0.0366± 0.0069	0.0084± 0.0049	0.0093± 0.0015	0.3347± 0.0117	0.0166± 0.0040	0.0060± 0.0009
1-2D8	0.0441± 0.0012	0.0417± 0.0094	0.0185± 0.0095	0.0235± 0.0212	0.0537± 0.0250	BDL	BDL	BDL	BDL	0.0398± 0.0083	0.0262± 0.0144	0.0352± 0.0065	0.0418± 0.0161	0.0310± 0.0624	0.0488± 0.0077	0.0023± 0.0016	0.0054± 0.0011	0.3513± 0.0416	0.0075± 0.0007	0.0051± 0.0005
1-2F2	0.0454± 0.0070	0.0303± 0.0043	0.0190± 0.0076	0.0125± 0.0132	0.0583± 0.0458	BDL	BDL	BDL	BDL	0.0594± 0.0146	0.0429± 0.0138	0.0395± 0.0164	0.0504± 0.0161	0.0410± 0.0169	0.0383± 0.0046	0.0075± 0.0035	0.0055± 0.0007	4.9122± 0.1583	0.0166± 0.0005	0.0091± 0.0018
1-2B3	0.0767± 0.0283	0.0547± 0.0096	0.0471± 0.0103	0.0333± 0.0276	0.0809± 0.0225	BDL	BDL	BDL	BDL	0.0882± 0.0593	0.0438± 0.0218	0.0633± 0.0187	0.0838± 0.0331	0.0486± 0.0407	0.1248± 0.0586	0.0060± 0.0026	0.0114± 0.0039	0.0224± 0.0090	0.0215± 0.0073	0.0100± 0.0057
1-1C7-4	0.0705± 0.0070	0.0612± 0.0043	0.0385± 0.0076	0.0258± 0.0132	0.0791± 0.0458	BDL	BDL	BDL	BDL	0.0585± 0.0146	0.0562± 0.0138	0.0416± 0.0164	0.0815± 0.0161	0.0492± 0.0621	0.1063± 0.0046	0.0061± 0.0034	0.0097± 0.0007	0.0467± 0.0058	0.0467± 0.0081	0.0048± 0.0018
1-G1	0.0791± 0.0289	0.0584± 0.0310	0.0388± 0.0212	0.0498± 0.0293	0.1284± 0.0597	BDL	BDL	BDL	BDL	0.0852± 0.0090	0.0777± 0.0349	0.0916± 0.0275	0.1243± 0.0563	0.0580± 0.0557	0.1576± 0.0334	0.0059± 0.0035	0.0130± 0.0047	0.0157± 0.0070	0.0289± 0.0218	0.0145± 0.0105
4-1H1	0.5826± 0.0683	0.1015± 0.0169	0.1177± 0.0325	0.0795± 0.0299	0.1208± 0.0216	BDL	BDL	BDL	BDL	0.0981± 0.0088	0.1158± 0.0056	0.1280± 0.0222	0.1755± 0.0294	0.0515± 0.0107	0.1492± 0.0093	0.1176± 0.0068	0.0222± 0.0008	0.0399± 0.0339	0.0247± 0.0135	0.0202± 0.0008
4-1E1-4	0.2010± 0.0343	0.1056± 0.0250	0.1002± 0.0120	0.0752± 0.0391	0.1288± 0.0033	BDL	BDL	BDL	BDL	0.0945± 0.0132	0.0940± 0.0037	0.0968± 0.0195	0.2333± 0.0910	0.0494± 0.0243	0.1403± 0.0266	0.1343± 0.0088	0.0289± 0.0115	0.0416± 0.0211	0.0516± 0.0109	0.0266± 0.0051
4-F10-1	0.0288± 0.0018	0.0152± 0.0040	0.0108± 0.0077	0.0082± 0.0009	0.0639± 0.0126	BDL	BDL	BDL	BDL	0.0339± 0.0110	0.0302± 0.0108	0.0265± 0.0180	0.0371± 0.0180	0.0233± 0.0053	0.0399± 0.0149	0.0124± 0.0029	0.0046± 0.0020	0.0089± 0.0010	0.0142± 0.0041	0.0028± 0.0015
2-2H5-1	0.0725± 0.0044	0.0525± 0.0026	0.0195± 0.0146	0.0207± 0.0214	0.0764± 0.0460	BDL	BDL	BDL	BDL	0.0447± 0.0104	0.0390± 0.0297	0.0505± 0.0138	0.0502± 0.0103	0.0438± 0.0143	0.1332± 0.0217	0.0177± 0.0016	0.0053± 0.0006	0.0301± 0.0036	0.0112± 0.0042	0.0040± 0.0008
1-2F8	0.0572± 0.0194	0.0444± 0.0285	0.0386± 0.0183	0.0137± 0.0151	0.0367± 0.0285	BDL	BDL	BDL	BDL	0.0339± 0.0140	0.0445± 0.0077	0.0457± 0.0152	0.0717± 0.0484	0.0303± 0.0028	0.5382± 0.1844	0.1388± 0.0097	0.0149± 0.0041	BDL	0.6643± 0.1685	0.0062± 0.0028
1-2D3-3	0.0705± 0.0432	0.0571± 0.0183	0.0495± 0.0019	0.0187± 0.0112	0.0552± 0.0302	BDL	BDL	BDL	BDL	0.0389± 0.0179	0.0434± 0.0094	0.0398± 0.0089	0.0570± 0.0307	0.0310± 0.0345	1.1269± 0.1167	0.2311± 0.0893	0.0240± 0.0102	7.3431± 2.6368	0.8642± 0.4177	0.0074± 0.0009
2-2F1	0.0421± 0.0375	0.0570± 0.0043	0.0325± 0.0246	0.0310± 0.0327	0.1025± 0.0320	BDL	BDL	BDL	BDL	0.0680± 0.0123	0.0682± 0.0278	0.0706± 0.0226	0.0992± 0.0553	0.0716± 0.0365	0.1973± 0.0917	0.0460± 0.0021	0.0210± 0.0126	0.0579± 0.1036	0.0711± 0.0257	0.0115± 0.0067
1-1C2	0.0624± 0.0102	0.0325± 0.0071	0.0218± 0.0118	0.0228± 0.0203	0.0571± 0.0450	BDL	BDL	BDL	BDL	0.0479± 0.0057	0.0454± 0.0190	0.0431± 0.0129	0.4611± 0.7128	0.0245± 0.0584	0.0705± 0.0171	0.0058± 0.0029	0.0063± 0.0012	0.2777± 0.0482	0.0171± 0.0018	0.0058± 0.0014
4-2E10	0.0667± 0.0127	0.0543± 0.0093	0.0457± 0.0123	0.0509± 0.0280	0.0753± 0.0257	0.2252± 0.0345	BDL	BDL	BDL	0.0524± 0.0213	0.0597± 0.0342	0.0706± 0.0465	0.0826± 0.0248	0.0337± 0.0107	0.0984± 0.0167	0.0801± 0.0036	0.0124± 0.0026	0.0153± 0.0087	0.0081± 0.0034	0.0104± 0.0037

Nb70	0.2631± 0.0490	0.2324± 0.0656	0.2382± 0.0427	0.1737± 0.0742	0.3696± 0.0143	0.4220± 0.1282	3.6383± 0.3146	0.2045± 0.0844	0.0798± 0.0258	0.2141± 0.0357	0.3450± 0.0434	0.3427± 0.0172	0.3696± 0.3274	0.1747± 0.0378	0.2135± 0.0656	0.0260± 0.0056	0.0220± 0.0053	0.0793± 0.1198	0.0541± 0.0068	0.0170± 0.0063
Nb3	0.2308± 0.1274	0.1603± 0.0774	0.1825± 0.0615	0.1044± 0.0173	0.2494± 0.0015	3.0691± 0.3757	BDL	BDL	BDL	0.2012± 0.0328	0.3075± 0.0085	0.2652± 0.0001	0.2494± 0.5799	0.1444± 0.0728	0.1765± 0.0400	0.0176± 0.0083	0.0157± 0.0050	0.0259± 0.0180	0.0227± 0.0057	0.0102± 0.0002
Nb7	0.2571± 0.1289	0.2417± 0.0046	0.2016± 0.0265	0.1500± 0.0085	0.3895± 0.1352	1.1550± 0.1353	BDL	BDL	BDL	0.2887± 0.0385	0.3579± 0.1410	0.2272± 0.1970	0.3895± 0.5257	0.1633± 0.0228	0.2269± 0.0251	0.0356± 0.0497	0.0256± 0.0094	0.0231± 0.0183	0.0354± 0.0174	0.0287± 0.0188
3-2A2-4	0.0418± 0.0044	0.0236± 0.0048	0.0407± 0.0248	0.0201± 0.0121	0.0208± 0.0105	0.0317± 0.0124	0.0467± 0.0166	0.1700± 0.0936	0.1532± 0.0580	0.0261± 0.0050	0.0296± 0.0173	0.0336± 0.0104	0.0540± 0.0149	0.0385± 0.0738	0.0420± 0.0064	0.0156± 0.0108	0.0339± 0.0275	0.5007± 0.0605	0.0503± 0.0175	0.0373± 0.0148
4-1A10-1	0.0365± 0.0037	0.0205± 0.0089	0.0329± 0.0549	0.0294± 0.0051	0.0286± 0.0050	0.0095± 0.0059	0.0417± 0.0155	0.0205± 0.0328	0.0472± 0.0033	0.0368± 0.0431	0.0391± 0.0268	0.0507± 0.0026	0.0427± 0.0105	0.0386± 0.0127	0.0371± 0.0042	0.0583± 0.0015	0.0153± 0.0052	0.3133± 0.0639	0.0373± 0.0254	0.0310± 0.0061
4-1A10-4	0.0425± 0.0097	0.0264± 0.0065	0.0270± 0.0142	0.0513± 0.0111	0.0318± 0.0065	0.0469± 0.0214	0.1482± 0.0813	0.1762± 0.0935	0.2686± 0.1254	0.0242± 0.0212	0.0285± 0.0161	0.0309± 0.0024	0.0883± 0.0354	0.0325± 0.0043	0.0592± 0.0013	0.0395± 0.0065	0.0414± 0.0018	0.3475± 0.0882	0.0983± 0.0193	0.0422± 0.0205
1-E1-1	0.0226± 0.0041	0.0100± 0.0016	0.1176± 0.0035	0.1353± 0.0335	0.0235± 0.0207	BDL	3.5501± 1.8514	1.3235± 1.4091	BDL	0.0081± 0.0046	0.0275± 0.0130	0.0239± 0.0112	0.0073± 0.0045	0.0105± 0.0139	0.0220± 0.0015	BDL	0.0074± 0.0024	BDL	BDL	BDL
1-C2	0.0052± 0.0024	0.0060± 0.0005	BDL	10.3595 ±0.0002	0.0043± 0.0002	0.4458± 1.9124	BDL	BDL	BDL	2.3914± 1.9506	BDL	BDL	0.2853± 0.1212	0.0174± 0.0332	0.0046± 0.0004	BDL	0.0031± 0.0016	BDL	BDL	2.8394± 0.9178
1-C3-4	0.0193± 0.0017	0.0013± 0.0005	BDL	14.6794 ±4.3574	0.0114± 0.0077	BDL	BDL	BDL	BDL	BDL	BDL	BDL	3.5508± 2.8097	0.0151± 0.0035	0.0031± 0.0013	BDL	0.0046± 0.0032	BDL	BDL	2.4327± 2.1685
1-2D3-1	0.0100± 0.0039	0.0042± 0.0021	BDL	BDL	0.0921± 0.0689	BDL	BDL	BDL	BDL	0.2781± 0.0179	BDL	BDL	0.2979± 0.1271	0.1017± 0.0481	0.0098± 0.0049	BDL	0.0099± 0.0073	BDL	BDL	4.2055± 2.2157
3-2D7-2	0.0134± 0.0060	0.0411± 0.0272	BDL	9.4262± 5.2642	0.3810± 0.3591	BDL	BDL	BDL	BDL	0.0528± 0.2644	BDL	BDL	BDL	1.5963± 0.2644	0.0116± 0.0008	BDL	0.0058± 0.0010	BDL	BDL	BDL
1-1G9	0.0136± 0.0039	0.0019± 0.0003	1.7200± 0.4175	1.4834± 0.1644	0.0061± 0.0049	BDL	BDL	BDL	BDL	0.0049± 0.0022	0.0993± 0.0238	0.1299± 0.0490	0.0031± 0.0016	0.0017± 0.0001	0.0133± 0.0005	BDL	0.0046± 0.0029	BDL	BDL	1.3705± 2.6586

Table S2. Data collection and refinement statistics

	1-2C7-SARS-CoV-2 SA-RBD	Nb70-1F11 Fab- SARS-CoV-2 WT-RBD	Nb70-SARS-CoV-1 WT-RBD	3-2A2-4-SARS-CoV-2 WT-RBD
**Data collection**				
Space group	I23	$P2_12_12_1$	$P3_121$	$P4_12_12$
Cell dimensions				
a, *b*, *c* (Å)	143.619 143.619 143.619	94.629 95.47 104.139	108.413 108.413 94.119	89.168 89.168 129.154
α, β, γ (°)	90, 90, 90	90, 90, 90	90, 90, 120	90, 90, 90
Resolution (Å)	50-1.8(1.864-1.8)	50-2.4(2.486-2.4)	50-2.4(2.486-2.4)	50-2.4 (2.488-2.4)
R_{sym} or R_{merge}	0.172 (2.643)	0.213 (1.249)	0.135 (2.269)	0.152 (2.555)
$I / \sigma I$	33.27 (1.57)	17.18 (1.74)	25.45 (1.10)	23.43 (1.54)
Completeness (%)	99.93 (100)	99.54 (99.89)	99.77 (99.84)	97.37 (83.09)
Redundancy	38.8(31.2)	12.2 (8.5)	17.9 (14.3)	22.1 (20.9)
**Refinement**				
Resolution (Å)	25.39-1.8	45.62-2.4	33.24-2.4	28.33-2.4
No. reflections	45590 (4537)	37381 (3676)	25337 (2493)	20419 (1695)
R_{work} / R_{free}	17.99/20.97	19.53/24.36	19.98/23.78	22.96/28.74
No. atoms				
Protein	323	745	375	315
Ligand/ion	39	14	28	49
Water	225	216	51	30
B-factors				
Protein	44.17	43.35	64.96	69.22
Ligand/ion	67.20	52.62	103.73	69.84
Water	46.31	62.66	61.38	77.73
R.m.s. deviations				
Bond lengths (Å)	0.007	0.008	0.008	0.010
Bond angles (°)	0.87	0.99	1.01	1.25

Table S3. Contact residues of the Nbs-RBD interfaces

Nb70	SARS-CoV-1 RBD	Nb70	SARS-CoV-2 RBD	1-2C7	SARS-CoV-2 RBD	3-2A2-4	SARS-CoV-2 RBD
Q1	G400	Q1	G413	Q1	K378	S29	D364
	T402	T28	D427	L31	Y369		V367
T28	D414		D428		P384	L31	L335
	D415	R31	Y380	R57	A372		V362
R31	Y367		G381	R100	T376		A363
	G368		D428		F377		D364
	D415		F429		K378		P527
	F416		T430	P101	Y369	V33	L335
	M417	Y32	Y380		F374	L53	T333
Y32	Y367		P412		S375		N334
	P399	E52	S383		F377		L335
E52	S370	W53	G381	S102	Y369		V362
W53	V369		V382		S371	D54	T333
	G368		S383		A372	E98	L335
	S370	G100	C379		S373	N99	V367
G100	C366		Y380		F374	G100	A363
	Y367	N101	K378		F377		D364
N101	K365		C379	A103	Y369		V367
	C366		G381		N370	G101	L335
	G368		V382		S371		C336
	V369		P384	H104	N370		F338
	A371	Q102	F377		S371		G339
Q102	F364		K378		A372	F102	F338
	K365		P384	Y105	S371		G339
	A371	Y103	Y369		A372		F342
Y103	Y356		F377		F374		N343
	F364		P384		S375		D364
	A371	Y104	S371	Y109	V503		Y365
	T372		A372	T111	V503		L368
Y104	T359		F374		G504	F103	F342
	F361	Y114	R408	E112	S375		N343
N112	R395	D115	K378		Y508		V367
E113	R395		Y380	D114	K378		S371
Y114	R395		R408				F374
D115	K365		A411			Y104	G339
	Y367		Q414				N343

	R395<br>A398<br>Q401	F116	P412<br>G413<br>Q414
F116	P399<br>G400<br>Q401	W117	R408

Table S4. Cryo-EM data collection statistics

	SARS-CoV-2 Omicron spike glycoprotein in complex with three nanobody 3-2A2-4 (EMDB-33923)
**Data collection and processing**	
Magnification	81000
Voltage (kV)	300
Electron exposure (e–/Å2)	50
Defocus range (μm)	1.5-1.8
Pixel size (Å)	1.0979
Symmetry imposed	C1
Initial particle images (no.)	947,196
Final particle images (no.)	106.254
Map resolution (Å)	3.29
FSC threshold	
Map resolution range (Å)	5-3.29
